# T-Cell Lymphoblastic Lymphoma in a Child Presenting as Rapid Thyroid Enlargement

**DOI:** 10.1155/2014/368590

**Published:** 2014-07-24

**Authors:** Shintaro Yoshihara, Muneo Nakaya, Tomoya Ichikawa

**Affiliations:** ^1^Department of Otolaryngology-Head and Neck Surgery, Tokyo Metropolitan Children's Medical Center, Fuchu, Tokyo 183-8501, Japan; ^2^Department of Otolaryngology-Head and Neck Surgery, Tokyo Metropolitan Tama Medical Center, Fuchu, Tokyo 183-8524, Japan

## Abstract

The majority of lymphomas of the head and neck in children present as an enlarged cervical lymph node; however, malignant lymphoma arising from the thyroid gland is extremely rare. We report a case of a 12-year-old child who was admitted to our hospital because of a history of rapidly progressive anterior neck swelling. Histopathological studies revealed this case to be T-cell lymphoblastic lymphoma. We performed chemotherapy and the patient has kept recurrence-free survival for 18 months after the beginning of the treatment. This is the 2nd case of T-cell lymphoblastic lymphoma in the thyroid gland in a child.

## 1. Introduction

Non-Hodgkin's lymphoma (NHL) is one of the most common head and neck pediatric malignancies commonly involving cervical lymph node, salivary glands, larynx, sinuses, orbit, and extranodal lymphoid tissue of Waldeyer's ring [1]. Primary thyroid lymphoma (PTL) in children is extremely rare; thus, there have been only 8 English literatures reported [1–8]. Furthermore, this is the 2nd case when limited to the subtype T-cell lymphoblastic lymphoma (T-LBL).

T-LBL accounts for 30% of all pediatric NHL cases and shows many similarities with T-cell acute lymphoblastic leukemia (T-ALL). The primary site of disease and the degree of bone marrow involvement distinguish these two disease entities clinically. Even the subtle molecular and cytogenic differences indicate that T-LBL and T-ALL do not share an immunophenotypic and oncogenic profile; T-LBL is an aggressive NHL and frequently invades the central nerve system (CNS); therefore, the treatment for T-LBL should include intensified chemotherapy as is the case for treatment of T-ALL [9, 10].

Herein we report a case of a 12-year-old child with T-LBL arising from the thyroid gland and describe its process of diagnosis and treatment.

## 2. Case Presentation

**Figure 1:**
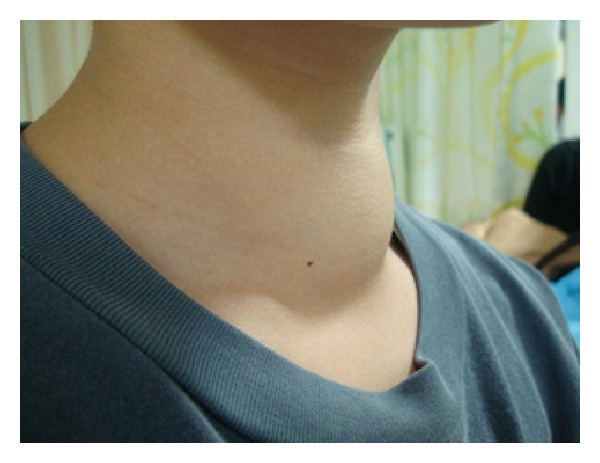
Anterior neck swelling without pain or redness.

**Figure 2:**
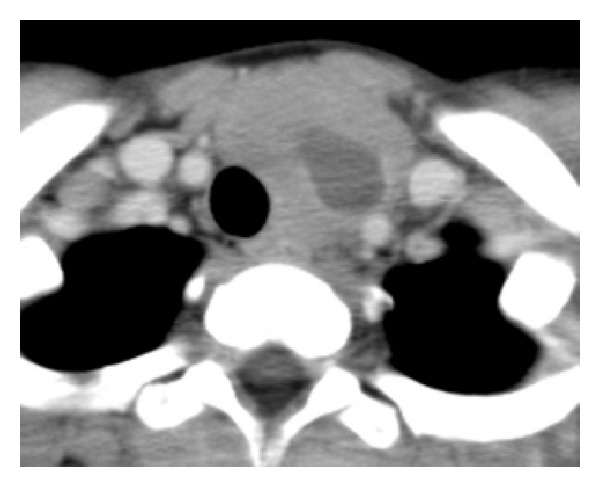
Initial axial computerized tomography scan. The thyroid mass in the inferior thyroid gland with a central necrotic area.

A 12-year-old Japanese child was admitted to our hospital because of a 3-day history of rapidly progressive anterior neck swelling (Figure 1). The mass was firm and nontender without pain or redness. Cervical lymphadenopathy was not recognized. His medical history was unremarkable and “B symptoms” were not obvious. There were no abnormalities in laboratory findings including thyroid functionality such as thyroid stimulating hormone (TSH) 1.47 *μ*IU/mL, free tri-iodothyronine (fT3) 4.64 pg/mL and free thyroxine (fT4) 1.13 ng/mL. Ultrasound examinations showed a large poorly defined tumor consisting of central numerous punctate lesion and peripheral hypoechoic area with increased vascularity. Calcification and cystic lesions were not present. Computerized tomography (CT) scan (Figure 2), magnetic resonance imaging (MRI), and scintigraphy using 201 Tl-Cl (69 MBq) suggested that the mass was a thyroid cancer in the inferior pole of the left thyroid gland with central necrosis. On the other hand, fine needle aspiration cytology of Papanicolaou stain revealed it to be class IV, suggesting malignant lymphoma (ML).

After discussion with haematologists and the patient's family, we decided to treat the patient with chemotherapy according to the specific subtype of histopathology and avoid total thyroidectomy to preserve thyroid function. We performed open biopsy under general anesthesia and excised a 1 cm^3^ specimen. After the surgery, TSH was 0.843 *μ*IU/mL, fT3 4.87 pg/mL, and fT4 1.14 ng/mL.

**Figure 3:**
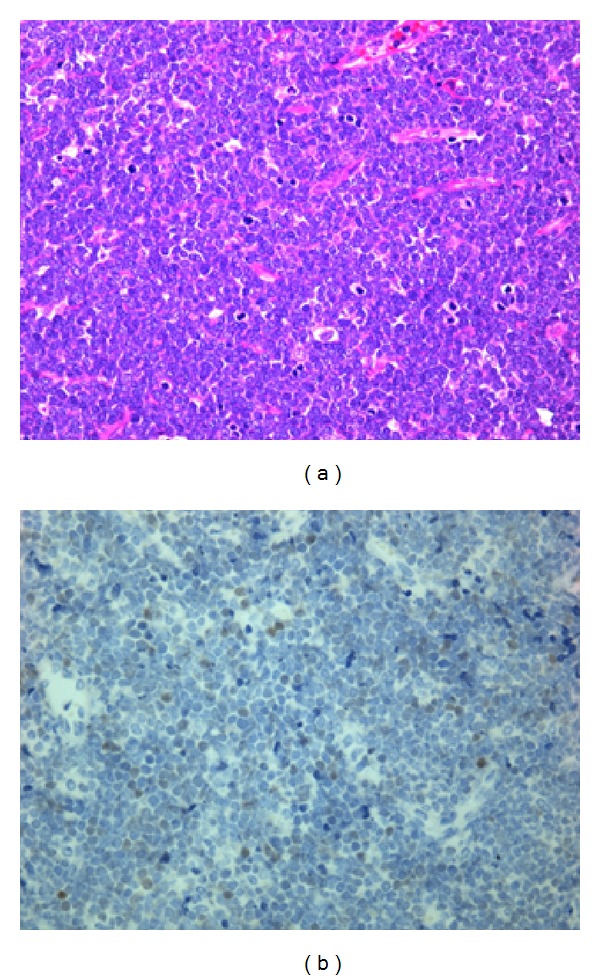
Histopathological examinations. T-cell lymphoblastic lymphoma. (a) The tumor was composed of medium sized lymphoblast with inconspicuous nucleoli (H&E; ×40). (b) The tumor cells were TdT positive.

Histopathological examinations including immunohistochemistry and flow cytometry showed CD45+, CD2+, CD3+, CD4+, CD5+, CD7+, CD8+, CD10+, and Terminal deoxynucleotidyl transferase (TdT)+; thus, the diagnosis was confirmed as T-LBL of the thyroid gland (Figures 3(a) and 3(b)). Additional investigations such as examinations of bone marrow and cerebrospinal fluid (CSF), MRI of the brain, and positron emission tomography (PET)-CT scan showed no evidence of metastasis; however, a CT scan indicated the possibility of swelling of the right tonsil. Hence we classified the present case as Murphy's classification stage II [11].

**Figure 4:**
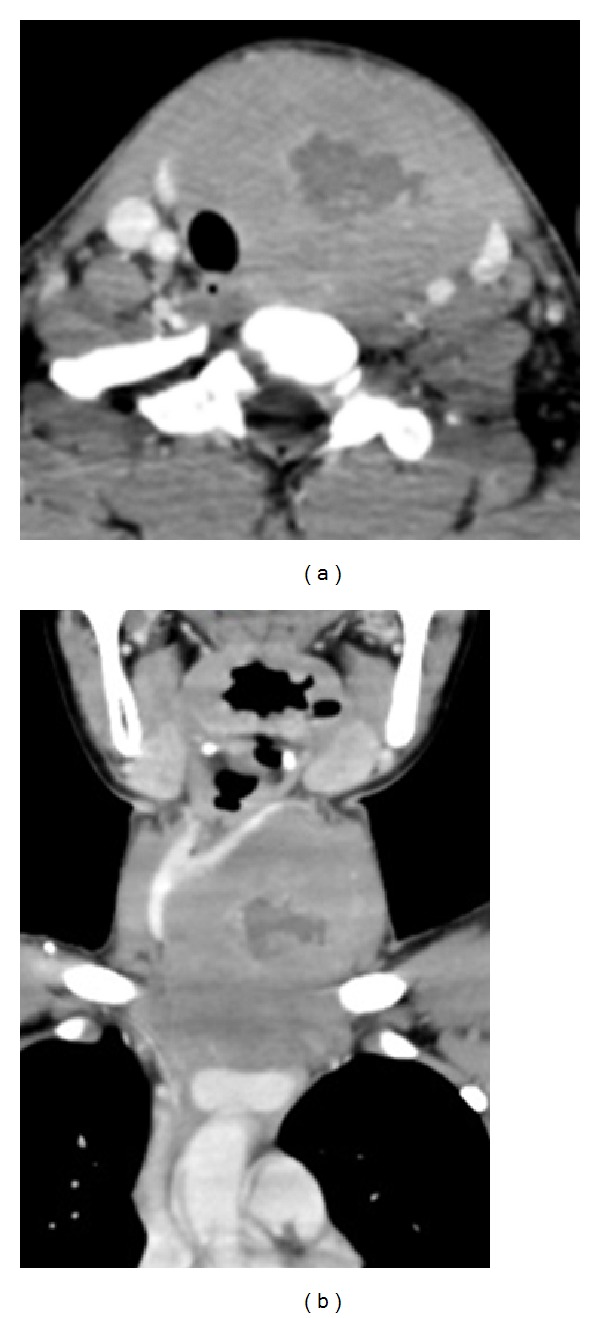
Axial (a) and coronal (b) CT scan showing the thyroid tumor larger than a month before (Figure 2).

The patient has been treated with chemotherapy in accordance with the protocol of Japan Pediatric Leukemia/Lymphoma Study Group (JPLSG). The protocol consists of prednisolone, vincristine, cyclophosphamide, daunorubicin, L-asparaginase, hydrocortisone, cytarabine, 6-mercaputopurine, and methotrexate. The thyroid mass had enlarged in a month from the patient's initial visit to the administration of chemotherapy (Figures 4(a) and 4(b)); the tumor showed rapid response for chemotherapy within 5 days, and a CT scan after the first phase of the treatment revealed a 90% decrease of the tumor.

A follow-up PET-CT scan after the fourth phase of the treatment showed that the tumor had totally disappeared. The patient has been treated with two years of maintenance chemotherapy consisting of 6-mercaputopurine and methotrexate after four cycles of chemotherapy were administered and has kept recurrence-free survival for 18 months after the beginning of the treatment.

## 3. Discussion

The classification of ML is presented by the 4th edition of the World Health Organization Classification of Tumors of Haematopoietic and Lymphoid Tissues published in 2008 [12]. In children, Burkitt's lymphoma and T-LBL represent 30–40%, respectively, and both DLBCL and anaplastic large cell lymphoma (ALCL) represent 10% of all NHLs [13].

Lymphoblastic lymphoma is derived from immature lymphocytes most of which are precursor T-cell origin. TdT is a specialized DNA polymerase expressed in immature lymphoid cells. The clinical distinction between T-LBL and T-ALL is based on the primary site of the disease and the degree of bone marrow involvement. With more than 25% of bone marrow involvement, the disease is classified as T-ALL. Because T-LBL is a high-grade lymphoma, CSF as well as bone marrow is involved in a higher rate. Furthermore, mediastinal lymph nodes frequently swell enormously and several complications such as breathing difficulty and superior vena cava syndrome often appear [14].

Before the 1970's a 5-year event-free survival was inferior to 10%; however, now it is well known that ALL-type regimen provides a successful control in T-LBL [15]. Several clinical trials have revealed factors to improve the prognosis of T-LBL, such as application of intensive ALL regimens, long term maintenance therapy, and intensive preventive care of CNS. CFS examination is required at diagnosis, and repeated intrathecal chemotherapy is needed whether malignant cells are revealed or not.

To the best of our knowledge, there have been 8 English literatures reported about pediatric PTL including only one case of a 9-year-old child with T-LBL [2]. Five cases have been in remission after chemotherapy. Because histopathological findings and staging of the disease are necessary for diagnosis and treatment plans, we should carefully perform strategic examinations with the knowledge of this rare malignant disease.
